# Angiotensin II receptor Neprilysin inhibitor (LCZ696) compared to Valsartan attenuates Hepatotoxicity in STZ-induced hyperglycemic rats

**DOI:** 10.7150/ijms.49373

**Published:** 2020-10-22

**Authors:** Faleh Alqahtani, Mohamed Mohany, Abdullah F Alasmari, Ahmed Z. Alanazi, Osamah M. Belali, Mohammed M Ahmed, Salim S Al-Rejaie

**Affiliations:** Department of Pharmacology and Toxicology, College of Pharmacy, King Saud University, P.O. Box 55760, Riyadh - 1145, Saudi Arabia.

**Keywords:** Hyperglycemic rats, LCZ696, valsartan, oxidative stress, inflammation, hepatic fibrosis

## Abstract

**Background and objectives:** Although diabetic-induced hepatotoxicity is less common, it can be included in the list of target organ pathologies associated with diabetes. This study aimed to investigate the potential therapeutic role of sacubitril/valsartan (LCZ696) in modulating oxidative and inflammatory injuries and liver fibrosis in STZ-induced hyperglycemic rats in comparison to valsartan alone.

**Materials and Methods:** Following the induction of diabetes using a single dose of streptozotocin (STZ), STZ-induced hyperglycemic animals were administered LCZ696 or valsartan for 6 weeks. Glucose, transaminases, lipid profile, tumor necrosis factor-alpha (TNF-α), interleukin 1 beta (IL-1β), and interleukin - 6 (IL-6), were estimated using the obtained serum. Oxidative stress biomarkers including thiobarbituric acid reactive substances (TBARS), glutathione (GSH), superoxide dismutase (SOD), catalase (CAT), glutathione peroxidase (GPx), and glutathione S-transferase (GST) were measured in the liver homogenate. Additionally, the levels of TNF-α, IL-1β, IL-6, and nuclear factor - kappa β (NF-κB) levels were estimated in hepatic tissue. To assess the general histopathological changes, harvested liver tissue was treated with hematoxylin and eosin or Masson's trichrome staining to detect fibrosis.

**Results:** STZ-induced hyperglycemic rats demonstrated high blood glucose, dyslipidemia, and significant elevation in hepatic transaminases, proinflammatory cytokines, NF-κB, lipid peroxidation, and hepatic fibrosis, with impairment in antioxidant enzymes. In STZ-induced hyperglycemic rats, the administration of LCZ696 ameliorated hyperglycemia, dyslipidemia, improved liver functions, and boosted antioxidants enzymes. Furthermore, LCZ696 therapy attenuated oxidation, inflammation, progression of liver injury, and hepatic fibrosis. LCZ696 was superior to valsartan in reducing AST, hepatic fibrosis, tissue IL-1β, TNF-α and NF-κB. In addition, compared with the valsartan group, LCZ696 significantly increased the antioxidant parameters such as GSH, SOD, CAT and GPx.

**Conclusion:** Collectively, our data demonstrated that LCZ696 could suppress the progression of diabetes-induced hepatic fibrosis, correlating with reduced oxidative stress, hepatic inflammation and NF-κB compared with valsartan alone.

## Introduction

Type 1 diabetes is a chronic autoimmune disorder with a lack in insulin, elevated blood glucose levels and ketoacidosis 1. Type 1 diabetes accounts for 5-10% of the global cases of diabetes affecting children and adolescents worldwide 2, indicating a steadily increasing incidence rate of about 3% annually 3. Uncontrolled hyperglycemia may lead to defects and dysfunctions in different body organ systems, including the eyes, kidneys, and nerves 4. Additionally, diabetic patients present a higher risk of developing multiple liver diseases, including non-alcoholic fatty liver disease, fibrosis, cirrhosis, hepatocellular carcinoma, and viral hepatitis 5. Furthermore, type 1 diabetes encompasses various hepatic disorders, including high levels of liver enzymes, accumulation of glycogen 6 and liver steatosis 7.

The incidence of diabetic complications is linked to both inflammation and oxidative stress 8, 9. Hyperglycemia and/or the oxidation of fatty acids leads to the excessive release of reactive oxygen species (ROS) and subsequent oxidative stress in diabetes mellitus 10. ROS overproduction can modify cellular functions and induce lipid peroxidation, inflammation, and cellular death in different tissues 11. Furthermore, it can also activate transcription factors involved in cell signaling such as NF-κB and the release of proinflammatory cytokines 12, thereby impairing insulin signaling, lipolysis, and hepatic glucose production 13. Therefore, attenuation of hyperglycemia and its associated oxidative stress and inflammation is considered an effective strategy for alleviating diabetes-induced liver damage.

Angiotensin AT1 receptor blockers have been extensively used to treat hypertension and reduce complications associated with diabetes 14. Furthermore, these blockers demonstrate beneficial effects in the treatment of chronic liver disease. Accordingly, in carbon tetrachloride-induced hepatic fibrosis, valsartan has a protective effect against liver damage 15 and reduce fibrosis in patients with hepatitis C 16.

LCZ696 belongs to a new class of drugs combining the angiotensin II receptor antagonist valsartan and the neprilysin inhibitor sacubitril. This compound has been found to attenuate cardiac hypertrophy, fibrosis, and vasculopathy in a rat model of chronic kidney disease 17, 18, and inhibits inflammation, oxidative stress and glomerulosclerosis in diabetic nephropathy rat model 19. Additionally, a clinical trial showed that LCZ696 in patients with mild to moderate hepatic impairment was safe and well tolerated 20. Regarding LCZ696 and liver protection, emerging data have confirmed that LCZ696 improves portal hypertension in portal hypertensive rats through downregulation of hepatic endothelin-1 21. Moreover, LCZ696 has been shown to improve measurements of liver function in patients with heart failure (HF) with reduced ejection fraction (HFrEF) 22. Based on the above studies, we hypothesized that administration of LCZ696 in an animal model of STZ-induced hyperglycemic rats may have a greater hepatoprotective effect compared with valsartan monotherapy. However, data elucidating LCZ696 impact on diabetes-induced hepatic injury are lacking and the effects of LCZ696 on liver function have not been assessed. We utilized this rat model that displays hyperglycemia-induced oxidative stress, inflammation and hepatic fibrosis to evaluate the impact of combined angiotensin receptor blocker (valsartan) and neprilysin inhibitor (sacubitril) on STZ-induced oxidative stress, inflammation, and hepatic fibrosis in comparison to valsartan alone.

## Materials and Methods

### Animals

Adult male Wistar rats (250-300 g) were collected from the Central Animal Facility, Pharmacy College, King Saud University, where they were maintained and monitored in a pathogen-free environment. In compliance with the National Institute of Health Guide for the Care and Use of Laboratory Animals, Institute for Laboratory Animal Research (NIH Publications No. 80-23; 1996), all experimental procedures, including euthanasia, were performed under the ethics approval number (SE-19-118). Prior to experimentation, all animals were acclimatized in polycarbonate cages in a well-ventilated room for 1 week. The animals were maintained at standard laboratory conditions (temperature of 23 °C, relative humidity of 55±5 and 12-h light/dark cycle), and provided food and water *ad libitum.*

### Experimental procedures and treatments

DM was induced in overnight fasted rats following a single intraperitoneal injection (60 mg/kg) of streptozotocin (STZ) dissolved in freshly prepared 0.1 M citrate buffer with pH 4.5, as previously described 23. Next, to prevent hypoglycemic shock, the rats were provided free access to a dextrose solution (5%) for 24 h. Blood sugar levels were tested (mg/dl) 2 days after the STZ injection using a glucometer (ACCU-CHEK ACTIVE, Roche, Germany). Animals with blood glucose levels reaching >250 mg/dl were considered diabetic and were included in the study.

Eighteen diabetic male rats were randomly divided into three groups (*n* =6) and six normal rats were used as controls: 1) Control rats received the vehicle, 2) STZ-induced hyperglycemic rats received the vehicle, 3) STZ-induced hyperglycemic rats treated with valsartan (31 mg/kg/day) orally, and 4) STZ-induced hyperglycemic rats treated with LCZ696 (68 mg/kg/day) orally. Valsartan (Tabuvan®) and LCZ696 (EntrestoTM) tablets were suspended in 0.5% carboxymethyl cellulose (CMC) and administered by oral gavage in a volume of 0.5 ml/100 g body weight of each animal. For both medications, dose selection was based on previous literature 24. During the experimental phase, the control and STZ groups were administered similar volumes of 0.5% CMC as the vehicle. Treatment started 2 weeks after the diagnosis of diabetes and continued for six consecutive weeks. After the treatment period, overnight fasted animals were anesthetized with ketamine (Hikma Pharmaceuticals, Jordan, 94 mg/kg)/xylazine (Laboratories Calier, Spain, 10 mg/kg) mixture. Blood samples were collected utilizing cardiac puncture and placed into clean tubes. Serum samples were obtained by centrifugation at 3,000 rpm (800 g) for 10 min and stored at -80 °C until analysis. After blood collection, the animals were immediately decapitated, whole liver was dissected and weighed, a small portion was immersed in 10% neutral formalin buffer (pH 7.4) for subsequent use in histopathological studies. The remaining liver samples were immersed in liquid nitrogen for a minute and then stored at -80 °C until analysis.

### Biochemical assays

Serum levels of glucose was assessed using a commercially available kit (Randox Laboratories Ltd., UK and SPI bio, France, Millipore, EZRMI-13K, respectively). Serum alanine aminotransferase (ALT) and aspartate aminotransferase (AST) were determined using diagnostic kits (Human Diagnostics Worldwide, Wiesbaden, Germany). Total cholesterol (TC), triglycerides (TG), low-density lipoprotein-cholesterol (LDL-C), and high-density lipoprotein-cholesterol (HDL-C) levels were estimated using commercially available diagnostic kits (Randox Laboratory Ltd, UK).

### Determinations of serum cytokines

Serum levels of cytokines including IL-1β, TNF-α, and IL-6 were estimated by using ELISA kits for rats (R&D Systems Inc., USA).

### Preparation of liver tissue homogenate

The harvested livers were cut into separate portions, rinsed in cold physiological buffer pH 7.4 (1:10, w/v), and homogenized using a homogenizer (ULTRA-TURRAX^®^ T 25, IKA; Werke, Germany). In order to remove cell debris, this homogenate was centrifuged at 1000 rpm for 10 min at 4 °C. After discarding the pellets and to obtain the post-mitochondrial supernatant, a portion of supernatant was further centrifuged at 12000 rpm for 20 min and total protein concentrations in livers were measured according to the Lowry assay 25 using bovine serum albumin as the standard.

### Assay for antioxidants and lipid peroxidation markers

In the liver homogenate, TBARS and GSH levels were measured using ELISA kits (Cayman Chemical Co., USA). In post-mitochondrial supernatants of liver samples, enzymatic activities of SOD, CAT, GP× and GST were measured using ELISA kits (R&D Systems Inc., USA).

### Assay of liver cytokines and NF-κB levels

Hepatic levels of proinflammatory cytokines (IL-1β, TNF-α, IL-6) and hepatic NF-κB were determined using ELISA techniques (Thermo Scientific, Rockford, IL, USA).

### Evaluation of hepatic fibrosis

Masson's trichrome (M-T) stained sections were used for the detection of collagen deposition. In 50 randomly selected fields, the area of fibrosis was determined at a 200× magnification using the following formula (% positive area = stained area / total area × 100) as described earlier 26.

### Statistical analysis

Data are presented as the mean and standard error (mean ± SEM) (*n*=6). One-way ANOVA was performed to test the significant differences between the different groups. Newman-Keuls multiple comparison test was employed as a post hoc test. Statistical analyses were performed using Graph-Pad Prism version 8 (GraphPad Software, Inc., La Jolla, CA, USA).

## Results

### Effects of LCZ696 and valsartan on serum biochemical measurements

**Figure 1:**
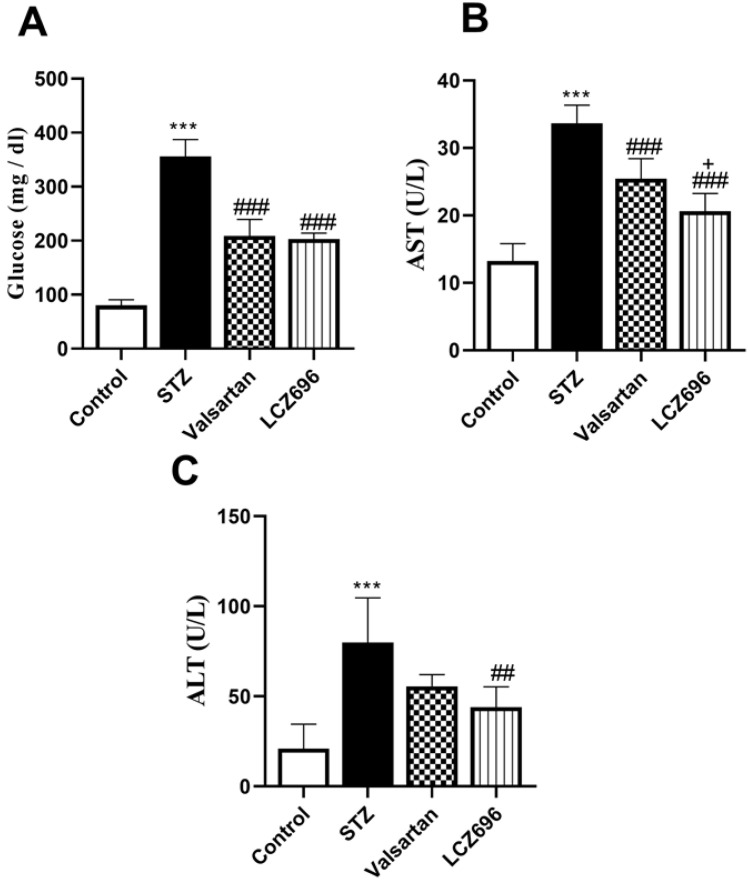
The effects of LCZ696 and valsartan on serum glucose, alanine aminotransferase (ALT), and aspartate aminotransferase (AST) in normal and STZ-induced hyperglycemic rats. Data were expressed as Mean ± SEM (*n*=6). The *p* values consider significant at ***P<0.001 compared with Control; ##*P*<0.01, ###*P*<0.001 compared with STZ-group and +*P*<0.05 compared with valsartan group (One-way ANOVA followed by Student Newman-Keuls as post hoc test).

Serum glucose levels were significantly (*p* < 0.001) elevated in the STZ-treated animals than the control group; these values were significantly lowered in STZ-induced hyperglycemic rats treated with LCZ696 (*p* < 0.001) and valsartan (*p* < 0.001) compared to the STZ-induced hyperglycemic rats (Figure 1A). AST and ALT levels were significantly (p< 0.001) elevated in the serum of STZ-induced hyperglycemic rats compared to the control group. However, treatment with LCZ696 or valsartan reversed these changes in liver enzymes compared with the untreated STZ-induced hyperglycemic rats (Figure 1B and 1C). As compared with the valsartan group, there was a significant decrease (*P*= 0.0304) in AST levels and a trend toward less ALT levels in the LCZ696 group.

### LCZ696 and valsartan mitigate hyperlipidemia in STZ-injected rats

**Figure 2:**
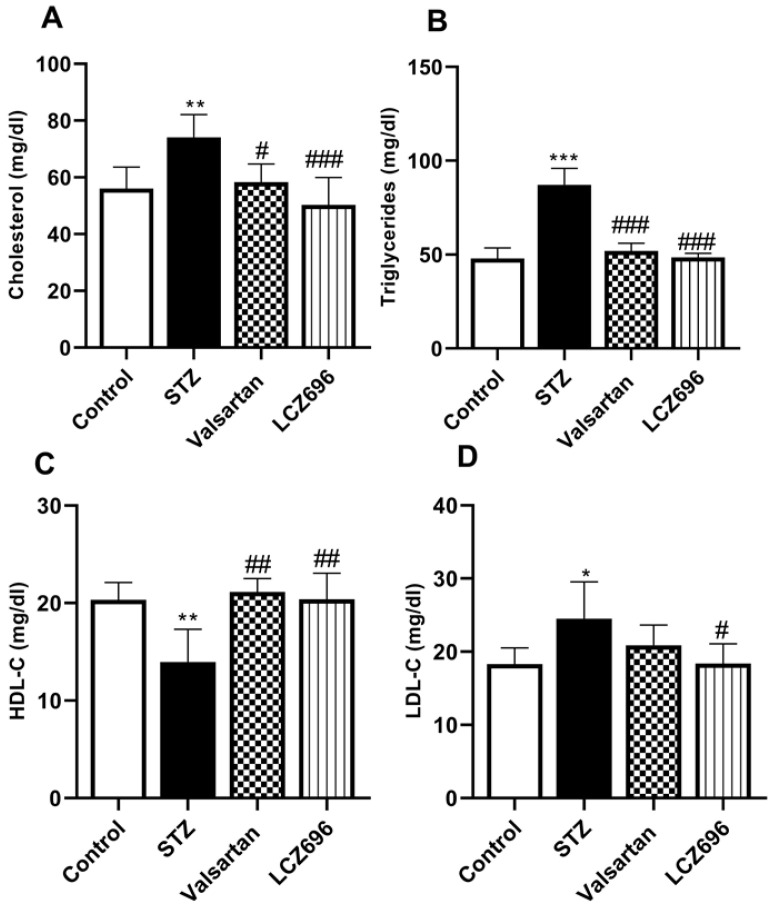
Influence of LCZ696 and valsartan on the serum lipid profile, including total cholesterol, triglyceride, high-density lipoprotein-C (HDL-C), and low-density lipoprotein-C (LDL-C) in normal and STZ-induced hyperglycemic rats. Data were expressed as Mean ± SEM (*n*=6) and analyzed using one-way ANOVA followed by Student Newman-Keuls as post hoc test. The *p* values consider significant when *P<0.05, **P<0.01 and ***P<0.001 compared with Control and #*P*<0.05, ##*P*<0.01, ###*P*<0.001 compared with STZ-group.

Serum lipids were determined to evaluate the anti-hyperlipidemic effect of LCZ696 and valsartan in STZ-induced hyperglycemic rats. The STZ-treated rats exhibited hypercholesterolemia (Figure 2A), hypertriglyceridemia (Figure 2B), and a marked increase (*p*<0.05) in the serum LDL-C levels (Figure 2D) relative to the control group; and a significant decrease in HDL-C (*p*<0.01) in the STZ group (Figure 2C), treatment with LCZ696 (68 mg/kg) and valsartan (31 mg/kg) increased HDL-C significantly (*p*<0.01) when compared with STZ-treated group. Additionally, in the STZ-induced hyperglycemic rats, therapy of LCZ696 (68 mg/kg) and valsartan (31 mg/kg) reduced serum cholesterol (*p*<0.001 and *p*<0.05, respectively), triglycerides (*p*<0.001), and LDL-C (*p*<0.05) compared to the STZ group. However, LDL-C reduction in valsartan-treated animals did not reach a statistically significant difference. There was a trend towards less cholesterol, triglycerides and LDL-C levels in the group treated with LCZ696 compared to the valsartan group.

### Impact of LCZ696 and valsartan on the circulating levels of IL-6, IL-1β, and TNF-α in STZ-induced hyperglycemic rats

**Figure 3:**
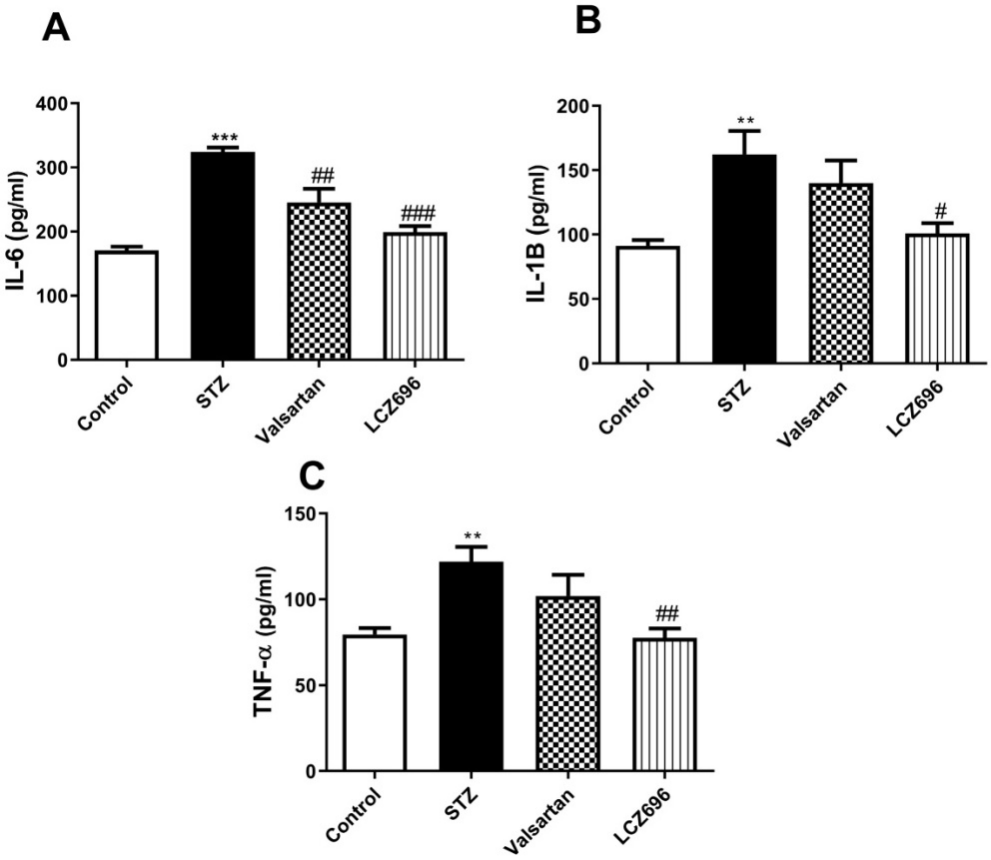
Effects of LCZ696 and valsartan on serum interleukin-6 (IL-6), interleukin-1β (IL-1β), tumor necrosis factor-α and (TNF-α), in normal and STZ-induced hyperglycemic rats. Data were expressed as Mean ± SEM (*n*=6) and analyzed using one-way ANOVA followed by Student Newman-Keuls as post hoc test. The p values consider significant when **P<0.01 and ***P<0.001 compared with Control and #*P*<0.05, ##*P*<0.01, ###*P*<0.001 compared with STZ-group.

Using ELISA, we assessed the serum profile of cytokines in experimental animals. On examining the serum levels of the proinflammatory cytokines, IL-6 (Figure 3A), IL-1β (Figure 3B), and TNF-α (Figure 3C) were significantly elevated in the STZ group compared to the control group. Treatment of STZ-induced hyperglycemic rats with LCZ696 significantly reduced the elevated levels of cytokines IL-6 (*P*<0.001), IL-1β (*P*<0.05) and TNF-α (*P*<0.01) compared to the STZ-treated group, unlike valsartan therapy which only attenuated levels of IL-6 (*P*<0.01). There was a trend towards lower proinflammatory cytokines in the LCZ696 group as compared with valsartan therapy.

### LCZ696 attenuates oxidative stress and enhances antioxidants enzymes in STZ-treated rats

**Figure 4:**
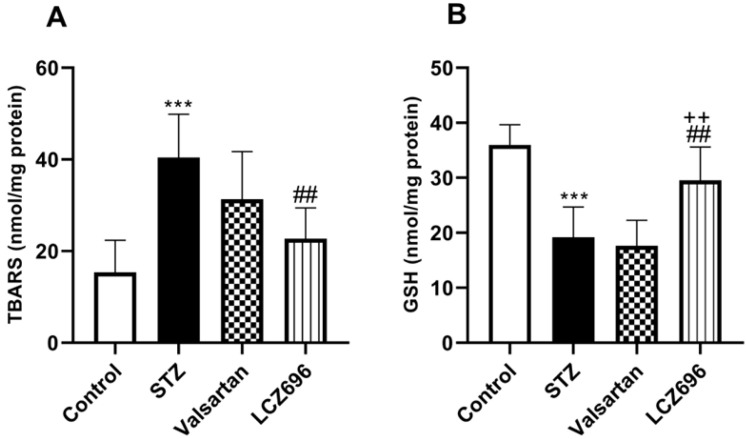
Effects of LCZ696 and valsartan on oxidative stress biomarkers including thiobarbituric acidreactive substances (TBARS) and glutathione (GSH) in hepatic cells. Data were expressed as Mean ± SEM (*n*=6). The *p* values consider significant at ***P<0.001 compared with Control; ##*P*<0.01, compared with STZ-group and ++*P*<0.01 compared with valsartan group (One-way ANOVA followed by Student Newman-Keuls as post hoc test).

**Figure 5:**
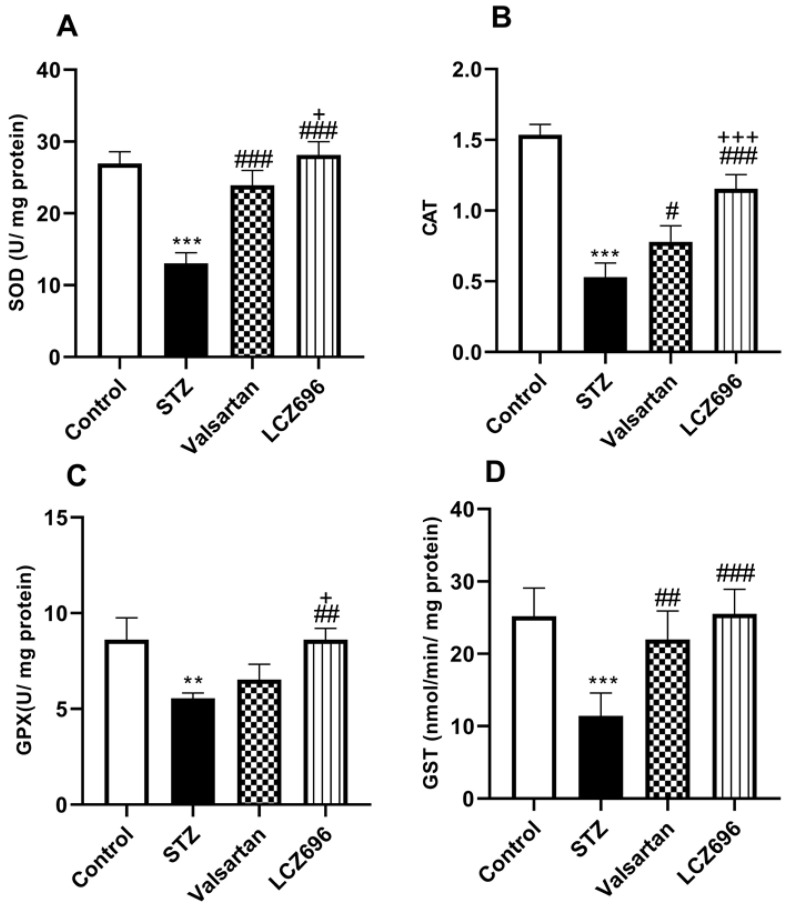
Effects of LCZ696 and valsartan on antioxidant enzyme activities (SOD, CAT, GPx, and GST) in the liver tissues of normal and STZ-induced hyperglycemic rats. Data were expressed as Mean ± SEM (*n*=6). The p values consider significant when **P<0.01, ***P<0.001 compared with Control; #*P*<0.05, ##*P*<0.01, ###*P*<0.001 compared with STZ-group and +*P*<0.05, +++*P*<0.001 compared with valsartan n group (One-way ANOVA followed by Student Newman-Keuls as post hoc test).

The ameliorative effects of LCZ696 and valsartan on oxidative stress were evaluated by assessing hepatic TBARS, GSH, SOD, CAT, GPx, and GST activities. STZ-injected rats exhibited hepatic oxidative stress, evidenced by a significant increase (*p*<0.001) in TBARS levels compared to the control animals (Figure 4A). Hepatic GSH (*p*<0.001) levels (Figure 4B) and enzymatic activities of SOD (*p*< 0.001, Figure 5A), CAT (*p*< 0.001, Figure 5B), GPx (*p*<0.01, Figure 5C), and GST (*p*<0.001, Figure 5D) were significantly reduced in STZ-treated rats compared to the control animals. Oral administration of LCZ696 prevented the increased levels of TBARS, enhanced hepatic GSH, and markedly enhanced SOD, CAT, GPx, and GST activities in STZ-induced hyperglycemic rats. However, valsartan therapy showed no significant improvements in hepatic TBARS, GSH and GPx relative to STZ-induced hyperglycemic rats, there was a significant increase in SOD, CAT, and GST levels. LCZ696 treatment for STZ-induced hyperglycemic rats was more effective than valsartan therapy in limiting oxidative stress and enhancing of antioxidant enzymes in particular GSH (*p*<0.01), SOD (*p*<0.05), CAT (*p*<0.001) and GPx (*p*<0.05).

### LCZ696 and valsartan suppress hepatic inflammation in STZ-induced hyperglycemic rats

**Figure 6:**
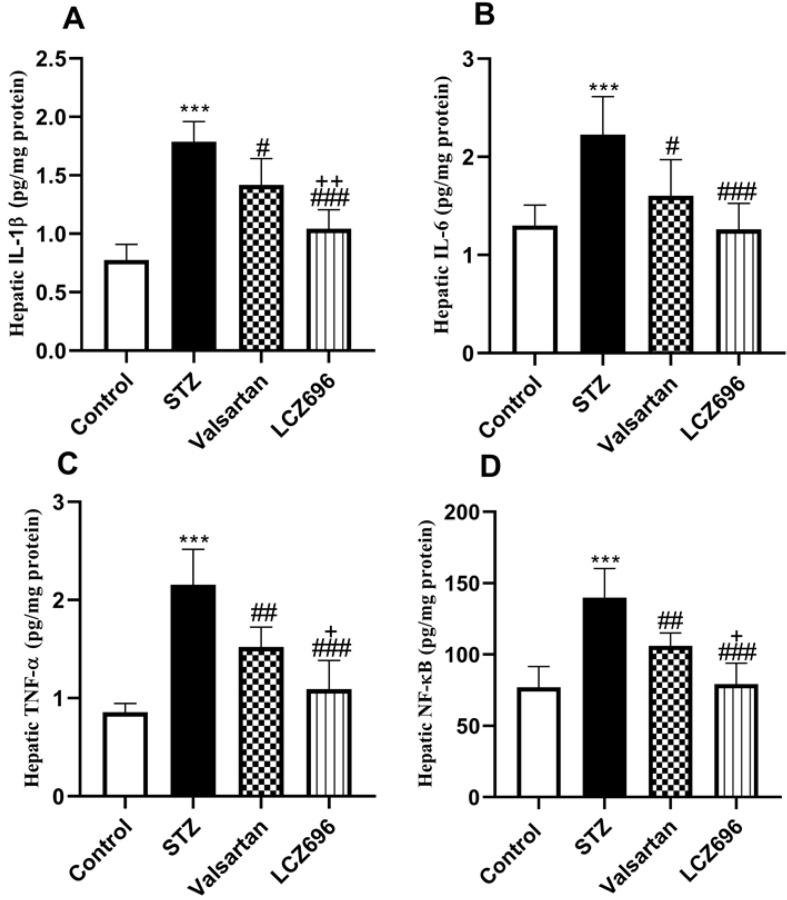
Effects of LCZ696 and valsartan on inflammatory biomarkers (IL-1β, IL-6, TNF-α, and NF-kB) in the liver homogenate of normal and STZ-induced hyperglycemic rats. Data were expressed as Mean ± SEM (*n*=6). The *p* values consider significant when ***P<0.001 compared with Control; #*P*<0.05, ##*P*<0.01, ###*P*<0.001 compared with STZ-group and +*P*<0.05, ++*P*<0.01 compared with valsartan group (One-way ANOVA followed by Student Newman-Keuls as post hoc test).

As shown in Figure 6, we examined whether LCZ696 and valsartan altered inflammatory responses in the liver of STZ-induced hyperglycemic rats. The hepatic levels of IL-1β, IL-6, TNF-α and NF-kB were significantly increased (*p*<0.001) in STZ-induced hyperglycemic rats compared to control animals. In STZ-induced hyperglycemic rats, treatment with LCZ696 and valsartan markedly inhibited the levels of IL-1β, IL-6, TNF-α, and NF-kB in hepatic tissue as compared to the STZ-treated group. But the inhibitory effect of treatment with LCZ696 for IL-1β (*p*<0.01), TNF-α (*p*<0.05) and NF-kB (*p*<0.05) in STZ-induced hyperglycemic rats was more effective when compared with the valsartan group.

### LCZ696 and valsartan prevent liver injury and fibrosis in STZ-induced hyperglycemic rats

**Figure 7:**
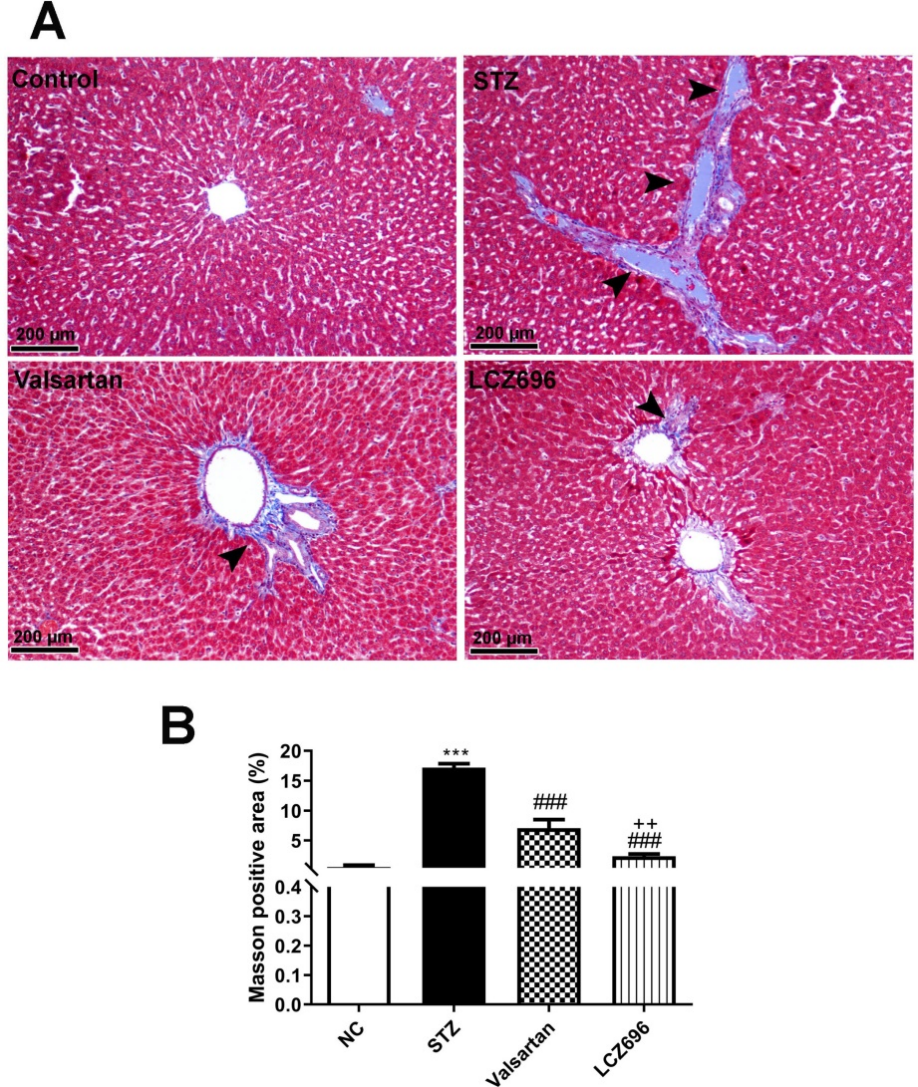
Masson's trichrome staining for collagen in liver sections from control and experimental groups. (a), Representative staining image showing the degree of fibrosis (head arrow). (b), Quantification of Masson's trichrome staining using ImageJ software. Data are presented as mean ± SEM. in each group. The *p* values consider significant when ***P<0.001 compared with Control; ###*P*<0.001 compared with STZ-group and ++*P*<0.01 compared with valsartan group (One-way ANOVA followed by Student Newman-Keuls as post hoc test).

Liver sections were stained with Masson's trichrome (M-T) to detect collagen deposition and assess the impact of LCZ696 and valsartan on hepatic fibrogenesis induced by STZ (Figure 7A). Compared with the control group, liver sections from the STZ-treated group demonstrated prominent blue staining in the fibrotic septa between lobules in particular in hepatic triad, suggesting a high level of collagen deposition. However, compared with the STZ group, LCZ696 and valsartan treatments significantly reduced the intensity of the livers areas positively stained using Masson's trichrome (*p*<0.001; Figure 7B). But the effect of treatment with LCZ696 was more effective when compared with the valsartan group.

## Discussion

Diabetes has serious effects on different body organs, especially the liver leading to the loss of its primary functions, resulting in multiple structural and functional abnormalities. Reportedly, most diabetic patients present with excessive glycogen accumulation, fibrosis, cirrhosis, fatty liver, and biliary disease 27. Here, we demonstrated that STZ-induced hyperglycemic rats exhibited high blood glucose, elevated liver enzymes, hyperlipidemia, production of proinflammatory cytokines, oxidative stress, and hepatic fibrosis. Using this model, we assessed the potential therapeutic impact of combining angiotensin II receptor blocker (valsartan) with neprilysin inhibitor (sacubitril) to alleviate diabetes induced-hyperlipidemia, oxidative stress, inflammation, and hepatic fibrosis. In addition, we evaluated the potential role of the NF-κB pathway in these injuries. The data obtained suggested that LCZ696 could substantially relieve hepatic fibrosis caused by STZ. Here, we proposed new information that LCZ696 could reduce oxidative stress, inflammation, and liver injury in rats with type 1 diabetes in comparison to valsartan.

Several reports have suggested the potential therapeutic role of angiotensin receptor-neprilysin inhibitors in the attenuation of diabetic complications 28, 29. In this study, STZ-induced hyperglycemic rats exhibited hyperglycemia and hepatic hyperlipidemia. This is consistent with previous studies 30, 31. In this study, our results indicated that valsartan possessed anti-hyperglycemic activity, this is supported by previous studies for example, Chan et al. 32 has reported that valsartan have anti-hyperglycemic effect via antagonism of the AT1 receptor resulting in increased use of glucose in peripheral tissue and reduced hepatic gluconeogenesis. In contrast, other studies have shown that valsartan and/or LCZ696 in STZ-induced hyperglycemic animals did not affect blood glucose levels 33, 34. These conflicting findings may be due to differences in the diabetic animal model, dosage and dosing frequency.

Interestingly, treatment of STZ-induced hyperglycemic rats with LCZ696 ameliorated hyperglycemia, liver dysfunction, and hyperlipidemia, indicating that, in the STZ-induced hyperglycemic rats, the combination of sacubitril and valsartan can provide a more favorable metabolic and hepatoprotective response compared to valsartan alone. Similarly, recent clinical data have explored the efficacy of LCZ696 in patients with diabetes, suggesting that this therapy demonstrated a superior glycemic profile 35. Additionally, simultaneous neprilysin inhibition and AT1‐receptor blockade reportedly control glucose and lipid metabolism in obese patients, including natriuretic peptides, bradykinin, endothelin‐1, and glucagon‐like peptide 1 36, thus elucidating the effect of LCZ696 on lipolysis and mitigating hyperlipidemia in STZ-treated rats.

Experimental and clinical studies have demonstrated that diabetes associated with hyperglycemia and that hyperlipidemia is linked to increased ROS, oxidative stress, and inflammation 37, 38. In this context, persistent hyperglycemia generates ROS, that contributes to oxidative damage of the macromolecules (lipids, carbohydrates, proteins, and nucleic acids) ultimately leading to tissue damage. Most strategies used to prevent hepatic damage from multiple insults are based on reduction of ROS and inflammation 39-41. In this study, STZ-induced hyperglycemic rats presented markers of elevated tissue oxidative injuries associated with increased malondialdehyde. In addition to these markers, a marked increase in the levels of the proinflammatory cytokines, as well as a deficiency in antioxidant enzymes, was observed. Consistently, other reports have revealed that hyperglycemia-induced elevated ROS levels reduced antioxidant enzyme levels 10 and increased proinflammatory cytokines 13. Moreover, increased plasma levels of TNF-α and IL-6 have been previously reported in STZ-treated rats 42. Previous studies have shown that IL-6 can inhibit glycogen synthesis 43, and TNF-α can decrease glucose uptake in hepatocytes and increase lipolysis 44, both of which can block hepatic insulin signaling.

The major findings of this study were that treatment with LCZ696 could halt the progression of hepatic fibrosis, oxidative damage, lipid peroxidation, and inflammation. LCZ696 treatments also reduced serum transaminases, cholesterol, and triglycerides. In addition, LCZ696 therapy boosted GSH and other antioxidants, inhibited hepatic NF-κB, and prevented hepatic injury in STZ-induced hyperglycemic rats. The blockage of the AT1 receptor by valsartan reportedly prevents the development of hepatic fibrosis via inhibition of TGF-β1 expression in type 2 diabetic rats 45. Reportedly, LCZ696 increases atrial natriuretic peptide (ANP) which inhibits the activation of the NF-KB pathway, reducing the development of ROS and cytokines 45, 46. This study elucidates that Attenuation of oxidative stress and inflammation with LCZ696 was instrumental in the superior suppression of hepatic fibrosis compared to valsartan alone. This improvement in hepatic architecture and functions following LCZ696 therapy may be attributed to the inhibition of neprilysin augmentation of natriuretic peptides (NPs). Furthermore, it has been reported that natriuretic peptide pathways participate in the suppression of cardiac fibrosis 47. Other studies have shown that collagen aggregation and cell proliferation are reduced in neprilysin deficient mice 48.

## Conclusions

In conclusion, the present study reported new information on the potential therapeutic impact of combined angiotensin II receptor antagonism and neprilysin inhibition using LCZ696 in diabetes-induced liver injury. Our targeted drug, LCZ696, prevented the pathological progression of hepatic fibrosis, associated with reducing oxidative stress, inflammation, and hepatic NF-κB. Therefore, LCZ696 might represent a potential new drug for the treatment of hyperglycemic rats-induced hepatotoxicity superior to valsartan treatment.
